# In Silico Analysis of Triamterene as a Potential Dual Inhibitor of VEGFR-2 and c-Met Receptors

**DOI:** 10.3390/jox14040105

**Published:** 2024-12-14

**Authors:** Stuart Lutimba, Baraya Saleem, Eiman Aleem, Mohammed A. Mansour

**Affiliations:** 1 Cancer Biology and Therapy Laboratory, School of Applied and Health Sciences, London South Bank University, London SE1 0AA, UK; stuart.lutimba@lsbu.ac.uk (S.L.); barayasaleem27@gmail.com (B.S.); abdelae3@lsbu.ac.uk (E.A.); 2 Biochemistry Division, Department of Chemistry, Faculty of Science, Tanta University, Tanta 31527, Egypt

**Keywords:** dual inhibitors, VEGFR-2, c-Met, molecular docking, molecular dynamics

## Abstract

The vascular endothelial growth factor receptor 2 (VEGFR2) and the hepatocyte growth factor receptor (C-Met) are critical receptors for signaling pathways controlling crucial cellular processes such as cell growth, angiogenesis and tissue regeneration. However, dysregulation of these proteins has been reported in different diseases, particularly cancer, where these proteins promote tumour growth, invasiveness, metastasis and resistance to conventional therapies. The identification of dual inhibitors targeting both VEGFR-2 and c-Met has emerged as a strategic therapeutic approach to overcome the limitations and resistance mechanisms associated with single-target therapies in clinical settings. Through molecular dynamics simulations and comparative docking analysis, we tested the inhibitory potential of 2,016 Food and Drug Administration (FDA)-approved drugs targeting VEGFR-2 and/or c-Met receptors. The results revealed that entacapone and telmisartan are potent and selective inhibitors for c-Met and VEGFR-2, respectively. Interestingly, triamterene was identified as a promising dual inhibitor, demonstrating specific and significant binding affinity to both proteins. Molecular dynamics simulations revealed key interactions between the identified compounds and critical residues in the catalytic domains of both VEGFR-2 (e.g., Lys868, Asp1028, Asp1046) and c-Met (e.g., Asp1204, His1202, Asp1222), providing insights into their mechanism of action. These findings underscore the therapeutic potential of triamterene in targeting multiple signaling pathways involved in cancer progression, metastasis and poor prognosis in patients. Our study provides a foundational framework for the development of novel anticancer compounds able to target multiple pathways in cancer. Further preclinical and clinical investigations are needed to validate the efficacy of these compounds in clinical settings and to test their ability to overcome resistance and improve patient outcome.

## 1. Introduction

Growth factor receptors (GFRs) play critical roles in normal cell processes including cell proliferation, angiogenesis and migration [1]. Among the GFRs, receptor tyrosine kinases (RTKs) are cell surface receptors that regulate various cellular processes including cell growth, differentiation and apoptosis [2]. Vascular endothelial growth factor receptor 2 (VEGFR-2) is a receptor tyrosine kinase that acts as a principal mediator of endothelial cell growth, proliferation and formation of new blood vessels [3]. These processes are fundamental for wound healing and embryonic development through promoting vascular permeability and formation of new blood vessels. Similarly, the mesenchymal–epithelial transition factor c-Met is a cell surface receptor but for another growth factor, hepatocyte growth factor (HGF) [4]. c-Met signaling promotes cell proliferation, migration and morphogenesis, which contributes to embryogenesis regulation and organogenesis. However, in various diseases, particularly cancer, VEGFR-2 and c-Met signaling pathways are dysregulated by overexpression or hyperactivation caused by gene amplifications or mutations, thus making these two receptors potential therapeutic targets [5]. Aberrant VEGFR-2 and c-Met signaling facilitate tumour growth, angiogenesis and metastasis of cancer cells, which are linked to aggressive tumour behaviour, poor clinical prognosis and resistance to therapy [3,6,7]. Consequently, dual-target tyrosine kinase inhibitors that simultaneously address both VEGFR and c-Met may offer more comprehensive benefits compared to inhibitors that selectively target either VEGFR or c-Met alone.

Various inhibitors of VEGFR-2 and c-Met receptors have been developed [8,9,10]. Inhibitors are categorized into three types [11], Type I ATP inhibitors that bind to the ATP-binding site, Type II inhibitors that stabilize the inactive conformation of the activation loop, and Type III inhibitors that form covalent bonds with cysteine residues, preventing ATP binding [12,13,14]. VEGFR-2 inhibitors such as sunitinib, sorafenib, cabozntinib and axitinib have shown efficacy in reducing tumour growth and angiogenesis in several types of cancers [9,15]. Similarly, c-Met inhibitors such as crizotinib, tivantinib and cabozantinib have demonstrated clinical significance by blocking HGF-mediated cell signaling and are currently used in clinical trials [16,17]. Despite these therapeutic advances, secondary mutations in target receptors, development of adaptive cellular responses and activation of alternative pathways have been reported to confer resistance to cancer cells against these inhibitors [9,14]. Treatment with a single inhibitor has proven to be both ineffective and limited in its impact, as drug resistance reduces efficacy and increases toxicity by allowing cancer cells to tolerate conventional chemotherapy. Consequently, the unchecked activity of these pathways can promote cancer cell proliferation, metastasis, and survival [2]. One of the mechanisms contributing to drug resistance involves the competition between intracellular ATP molecules and tyrosine kinase inhibitors [8]. This competition occurs at the ATP-binding site on receptor tyrosine kinases, activating downstream signaling pathway [11]. This resistance can be further exacerbated by the overexpression of protein tyrosine kinases (PTKs) due to mutations, which can lead to abnormal cell growth and carcinogenesis [16].

Preclinical studies have shown significant efficacy of dual target inhibitors (e.g., 3-(triazolo-thiadiazin-3-yl) indolin-2-one derivatives), which can effectively suppress tumour growth and angiogenesis even in cases where resistance has been developed due to monotherapy [8]. Dual inhibitors of VEGFR-2 and c-Met receptors represent a novel and promising therapeutic approach to overcome monotherapy resistance. By simultaneously targeting both proteins, various cancer-relevant cellular processes can be disrupted, thereby reducing the likelihood of developing alternative mechanisms and enhancing therapeutic efficacy. In this context, the screening of dual inhibitors through computational modeling represents a promising intervention for the development of potent cancer treatments. By targeting these specific receptors, the screening of dual target inhibitors aims to disrupt the signaling pathways associated with their overexpression, providing a potential strategy to overcome drug resistance and improve the efficacy of cancer treatment.

The structural composition of VEGFR-2 gene, which consists of 30 exons and 29 introns, codes for 1356 amino acids that fold into a stable structure comprising a signal peptide, a mature protein, an extracellular domain (ECD), a transmembrane domain (TMD), a Juxta Membrane Domain (JMD), and a catalytic tyrosine kinase domain (TKD) with an ATP-binding domain. The extracellular ligand-binding domain consists of seven immunoglobulin domains, and the intracellular segment contains two tyrosine kinase domains [18,19]. Upon binding with its ligand, VEGF-A, VEGFR-2 undergoes dimerization and autophosphorylation, initiating a phosphorylation signal cascade that enhances endothelial cell proliferation and migration. This process is crucial for angiogenesis and is often upregulated in cancer, contributing to tumour growth and metastasis [18].

c-Met gene consists of 21 exons and 20 introns to express c-Met protein, which includes alpha (32 kDa) and beta (120 kDa) subunits, with the hepatocyte growth factor (HGF) binding occurring at the Sema homology region (SEMA) domain [20]. This interaction leads to receptor homodimerization and phosphorylation of key residues (Y1234 and Y1235) within the intracellular tyrosine kinase domain, initiating a cascade of downstream signaling pathways [21]. Upon HGF binding, c-Met undergoes structural alterations, revealing a multi-substrate docking site that facilitates the binding of SH2 and SH3 domains, thereby activating various downstream signaling pathways. Previous studies have explored different strategies to inhibit c-Met signaling, including direct interference with c-Met and HGF signaling, blocking downstream signaling pathways, and inhibiting the tyrosine kinase activity of c-Met [20,21].

The current limitations of mono-target inhibitors of VEGFR-2 [19] and c-Met [20], including the development of resistance and incomplete inhibition of cancer progression, underscore the need for novel therapeutic dual-target inhibitors [8,13]. In the current study, using molecular docking and dynamic simulation, 2,016 Food and Drug Administration (FDA)-approved drugs were screened to identify compounds with dual inhibitory potential for both VEGFR-2 and c-Met receptors. Among these drugs, triamterene, a diuretic drug approved by the FDA, exhibited a novel dual inhibition of VEGFR-2 and c-Met receptors. Our computational analysis supports this dual inhibitory action of triamterene and highlights the drug as a potential candidate for further preclinical and clinical studies.

## 2. Methodology

### 2.1. Software Tools 

**Figure 1:**
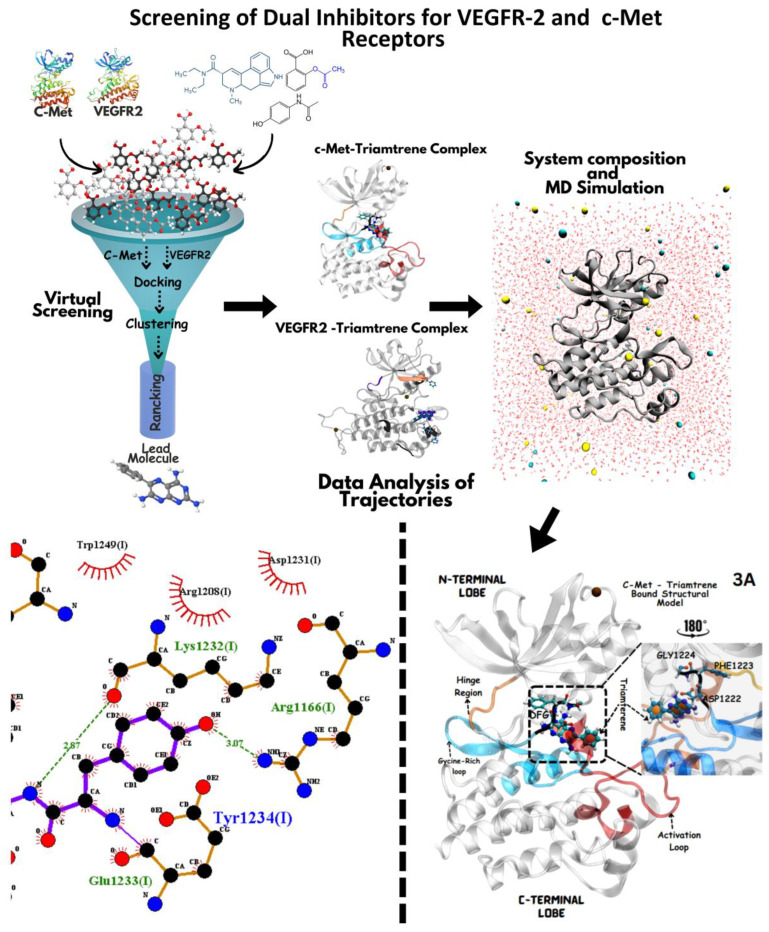
Computational workflow of repurposing FDA-approved drugs against the c-Met (PDB: 3LQ8) and VEGFR-2 (PDB: 3U6J) receptors. The process began with virtual screening (Step 1) of a drug library against these receptor active sites. Following this, ligand–protein complexes (Step 2) were prepared for molecular dynamics simulations, involving optimization, energy minimization, and equilibration using Gromacs and OpenMM (Step 3). These simulations were analyzed to evaluate stability, binding affinity, and interaction patterns. Finally, top candidates were prioritized based on binding affinity, clustering, and LOD scores, highlighting the most promising drug candidates for repurposing (Step 4).

The study utilized a diverse range of software tools for molecular modeling, simulation, and visualization tasks (Figure 1). Among these is our first developed-in-house molecular visualization platform, a pioneering web-based tool (MoLuDock Molecular Viewer) that enables rapid and interactive display of biological molecules in .pdb format. This platform can be accessed at https://lutimbastuart.github.io/Molecular_Carousel/ (accessed on 1 August 2024). Other tools utilized in the study included AutoDock [22] for predicting ligand–receptor interactions, Python for scripting and scientific computing, and MGLTools for molecular modeling utilities, including AutoDockTools [22]. Discovery Studio [23] provided a comprehensive suite for various molecular tasks, while UCSF Chimera [24] and PyMOL [25] facilitated interactive visualization of molecular structures. LigPlot+ [26] generated schematic diagrams of protein–ligand interactions, while Java Platform SE binary, Gromacs [27], and OpenMM [28] supported simulation and analysis tasks. CHARMM GUI [29] enabled setup and analysis of simulations, while PROCHECK [30] evaluated protein structure quality. VMD [31] served as a molecular visualization tool, and EPOS [32] likely aided in pocket analysis within proteins. Additionally, the ChemDraw [33] online server was used to create two-dimensional ligand structures.

### 2.2. Drug Library Preparation for c-Met and VEGFR-2 Screening

We employed an in-house computational drug library (MoLuDock lab) at London South Bank University (LSBU)) consisting of 2,016 FDA-approved drugs curated from the Zinc [34] and PubChem Databases. This library consisted of a variety of compounds, molecular scaffolds, functional groups, and therapeutic classes. The drug selection process for this library was designed to ensure chemical diversity while maintaining bioavailability and safety associated with FDA-approved compounds. Repurposing FDA-approved drugs offers significant advantages in clinical readiness and therapeutic development. These drugs have established safety profiles, reducing the risk of unforeseen adverse effects. Upon identifying a potent compound, drug development costs can be significantly reduced. Their pre-existing FDA approval facilitates a streamlined path to clinical trials for new indications, accelerating the translation of research into patient benefits. This strategy not only enhances drug discovery efficiency but also offers a cost-effective approach to developing novel cancer therapies, making treatments more accessible for patients.

Each molecule in the library was structurally optimized and standardized, ensuring compatibility with molecular docking and dynamic simulations. For this study, the library was deployed to identify potential binding drugs against C-MET and VEGFR-2 receptors, specifically targeting compounds with the capability to act as dual inhibitors. To optimize ligand–receptor interactions, we refined each ligand by adding hydrogen atoms where needed to ensure proper hydrogen bonding, stabilizing ligand configurations for docking [35].

We also accounted for charge distribution by introducing appropriate charges on polar groups to accurately reflect physiological conditions and enhance receptor-binding precision. This approach aimed to enhance the binding energy and interaction between the ligand and the protein. Additionally, each ligand’s ionization potential was characterized by assigning pKa values using Chemicalize [36], an online tool that calculates ionizable groups under physiological pH conditions. Post-optimization, Gromacs was employed to minimize the energy of each ligand structure, ensuring conformational stability and readiness for receptor interaction modeling (Figure 1).

### 2.3. VEGFR-2/c-Met Structural Refinement for Ligand Screening

**Figure 2:**
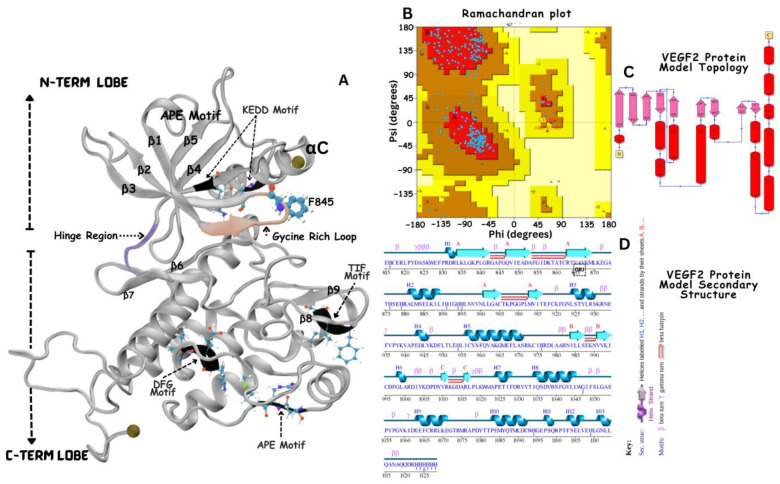
Structural model of the Apo state VEGFR-2 kinase domain. (**A**) represents the structural model of the VEGFR-2 kinase domain, featuring five anti-parallel beta sheets and one major alpha αC helix. The N and C lobes are connected by a hinge region, illustrated in purple. Key regulatory elements, including the TIF motif, KEDD motif, DFG motif, and APE motif, are highlighted in black ribbon. The Glycine rich loop is shown in orange (with residues 841–846). (**B**) represents the validation of the modeled structure is presented through Ramachandran plot analysis, confirming the stereochemical quality of the model. (**D**) presents the secondary structure and protein topology (**C**) is illustrated to emphasize the conformational integrity and spatial arrangement of the kinase domain.

**Figure 3:**
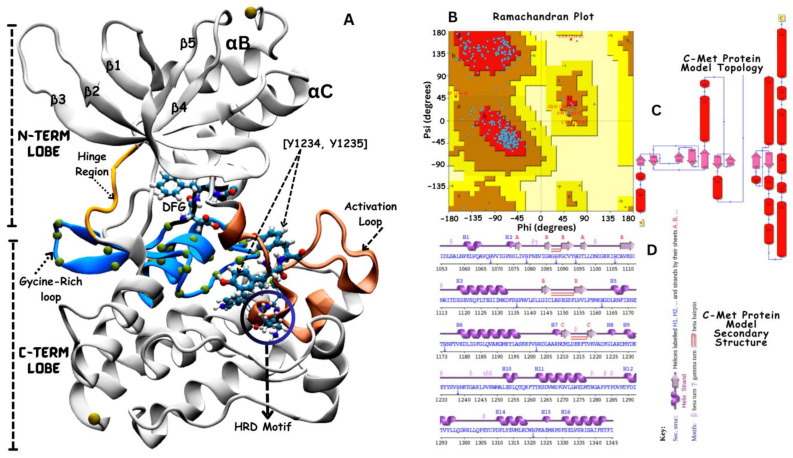
Structural model of the Apo state c-Met kinase domain (**A**). The yellow loop indicates the hinge region connecting the N-lobe and C-lobe. The blue cartoon represents the glycine-rich region loop, with each dot representing an amino acid. The orange region depicts the activation loop containing two critical tyrosine residues, Y1234 and Y1235 (shown in ball-and-stick), essential for kinase activation upon phosphorylation. Within the activation loop are the HRD motif, involved in catalytic activity, and the DFG motif, which plays a role in kinase activation and inhibition. The structure is validated by Ramachandran plot analysis (**B**), with the secondary structure (**D**) and protein topology (**C**) illustrating the hinge region (yellow), glycine-rich region (blue), activation loop (orange), and motifs HRD and DFG (shown in ball-and-stick).

The 3-dimension (3D) structure of VEGFR-2 (PDBID:3U6J [37]; resolution 2.15 Å) (Figure 2) and c-Met (PDBID: 3LQ8 [38]; resolution 2.02 Å), (Figure 3) proteins were acquired from the Protein Data Bank (PDB) database. The protein receptors contained other biological molecules such as co-crystalized ligands, water molecules and multiple protein chains.

The molecular processing workflow removes water molecules, adds any missing atoms, protonates titratable residues (such as histidine), minimizes energy, and validates the final protein model structure (see Figure 2B and Figure 3B). Each step is crucial in the refinement of the shape and stability of a protein to ensure that it precisely represents its most likely functional state in a biological environment. This preparation makes the protein more ready for correct interactions with the ligands, which optimizes its performance in further docking and molecular dynamics simulations.

### 2.4. Active Site Mapping via EPOS for Protein Inhibition

We employed a tool, the Ensemble of Pockets on Protein Surfaces, or EPOS [39], which screens for critical areas of the protein where inhibition could potentially occur. This approach employs a search based on pocket shape and features of the protein surface to locate a potential binding site. We defined pocket shapes using alpha complex analysis, an approach that quantifies the geometry of shape, to narrow the search to potential sites.

Having thus identified likely binding spots on the protein, we compared these with actual binding areas obtained from known protein structures. Using software called VMD, we compared our predictions by eye with real binding sites in these structures and found our identified areas to compare well with those observed experimentally.

### 2.5. VEGFR-2/c-Met Docking with AutoDock

AutoDock 4.2.6 [22], a renowned software for molecular docking, was obtained from the official website of The Scripps Research Institute, along with Python 3.12.2 [40] and MGLTools 1.5.4 [41]. Because of its shown accuracy in predicting protein–ligand interactions and its ability to handle large compound libraries efficiently, AutoDock 4.2.6 was selected for molecular docking. AutoDock is open-source, more accessible, and offers comparable reliability to proprietary programs like Glide and GOLD. Furthermore, it provides realistic and adaptable modeling of binding interactions due to its capacity to simulate partially flexible receptors and flexible ligands. The docking process involved manual docking of ligands and the kinase receptors (VEGFR-2/c-Met) individually to the protein using AutoDock as also used by other studies [42].

### 2.6. Protein/Ligand Preparation for AutoDock Docking

We first prepared the protein by adding specific hydrogen atoms and calculating charges necessary for the interaction modeling with a tool called AutoDock. This ensures that the protein is in the right form for docking. The ligand, which is the molecule that interacts with the protein, was also set up by identifying flexible parts that move during interactions. Both were saved in a format suitable for docking (PDBQT) and ready for simulations to observe how a protein and the ligand move and interact over time. Each step in this setup fine-tunes the structures such that the docking process can reliably model how the ligand would bind to the protein.

### 2.7. Grid Parameter Optimization for Precise Ligand Docking

Selecting the appropriate grid settings in MGLTools is vital for accurately directing the ligand to the binding sites of the receptors (c-Met and VEGFR-2) during molecular docking. We set the grid spacing to the default value of 0.375 Å, which provides a good balance between detail and computational efficiency. For the c-Met receptor, we defined the center of the grid box at coordinates x = 52, y = 70, and z = 62. The grid points were configured to measure 38.665 along the x-axis, 41.188 along the y-axis, and 29.419 along the z-axis. Similarly, for the VEGFR-2 receptor, we established the center grid box coordinates at x = 66, y = 60, and z = 72, with grid points measuring 53.136 along the x-axis, 46.056 along the y-axis, and 36.475 along the z-axis. These specific settings were chosen to ensure that the entire geometrics space of the active site for c-Met and VEGFR-2 was effectively covered. This approach helps enhance the accuracy of docking results by ensuring that the ligand can interact with all potential amino acids in the binding areas. Finally, we saved this grid information in a grid parameter file (GPF) for future reference, which allows for easy access and reproducibility in our simulations.

### 2.8. AutoGrid/AutoDock execution and Binding Energy Evaluation

The AutoGrid process was initiated by running the AutoGrid executable with the grid parameter files (GPFs) as input, in order to generate a grid log file (GLG). This step is very important because it sets up the computational environment required to accurately estimate how the ligand can dock into the binding site of the receptor. Having generated the grid, we ran AutoDock using docking parameter files (DPFs) as input. This generated a docking log file (DLG), which includes useful information such as free binding energies and inhibitory constants. We analyzed the results based on binding energies, and our focus was pointed at the identification of the lowest energy complex since it generally indicates the most favorable interaction between the ligand and the receptor. The lowest energy complex was then saved in PDB format for further examination. The structured approach guarantees that we would effectively evaluate potential drug candidates based on their binding efficiency, which is a very necessary stage within the drug discovery process.

### 2.9. Ranking Docked Ligand Poses for VEGFR-2 and c-Met via Virtual Screening

We performed in silico screening and evaluated docking results to explore new therapeutic applications for existing drugs. AutoDock Vina was utilized to dock 2,016 FDA-approved drugs onto the target protein (c-Met and VEGFR-2), allowing for 20 poses per drug. The docking box covered the entire protein structure with a 5 Å margin, and an exhaustiveness setting of 50 was chosen for accurate, yet timely results. Analysis of docking results focused on predicted binding affinity, distance metrics to target residues, Log odd score, and clustering of poses based on orientation. For binding affinity, both original values and normalized affinity were considered.

Distance measurements included the closest distance between drug atoms and the center of mass of target residues. Clustering is aimed at approximate entropy contribution, utilizing hierarchical clustering to group similarly oriented poses.

Two clustering methods were employed, COM-based clustering and adjacency-based clustering, with a cutoff of 4 Å. The resulting pose clusters were analyzed to identify the most favorable binding poses. Additionally, the evaluation of drug ranking methods, including LOD scores, was described. These methods aim to prioritize true binding poses among the numerous docked poses, thereby improving the efficiency and accuracy of drug screening.

### 2.10. LOD Scoring for Pose Ranking and Identification of Potential Ligands

The log-odds (LOD) score [43] measure was used to assess the likelihood of a sampled docking pose originating from a true binder versus a decoy. The LOD score is based on the concept of relative entropy, which compares the probability distributions (Equation (1)) of specific features for poses sampled from true binders and decoys. The LOD score was calculated for each sampled pose by considering the values of key features such as pose affinity, distance to the target site, and the size of pose clusters. These features are used to determine the likelihood of a pose being associated with a true binder or a decoy. The LOD score reflects how likely a given pose is to come from a true binder compared to a decoy. A higher LOD score indicates that a pose is more likely to be associated with a true binder, making it a valuable metric for ranking and prioritizing drugs in virtual screening and drug repurposing efforts. By incorporating the LOD score into the ranking process, this enhanced the accuracy and efficiency of drug screening by focusing on poses with higher probabilities of being true binders, thus facilitating the identification of potential candidates for further experimental validation and development.

(1) LOD Score ∑log ⁡PfTQfXPfFQfX

where

*X* represents a docked sampled pose.

*F* denotes the probabilities associated with the decoy poses, which are not true binders.

*T* denotes the probabilities associated with the true poses, which are expected to bind effectively to the target protein.

*f* denotes one of the specified features (e.g., pose affinity, distance to the target site, size of pose cluster).

*Q_f_ (X)* is the value of feature *f* for the pose *X*.

$P_{f}^{T}$ is the probability of distribution of feature *f* for docking poses sampled from the true binders.

$P_{f}^{F}$ is the probability distribution of feature *f* for docking poses sampled from decoys.

Equation (1) estimates the probability that a sampled pose is a true binder rather than a decoy; a higher score corresponds to a greater chance of being a true binder. The LOD score, which is calculated as relative entropy, reflects how likely a pose is to come from a true binder versus a decoy. The higher the LOD score, the stronger the likelihood of being a true binder. The 2,016 FDA-approved drugs were ranked by their best LOD score among 20 sampled poses.

### 2.11. MD Simulation of (VEGFR-2/c-Met) Ligand Complexes: Setup and Analysis

Molecular dynamics simulations were conducted using both GROMACS and OpenMM in order to leverage their complementing features. GROMACS was selected because of its excellent parallel computing capabilities, which enable effective simulation of big systems in a shorter amount of calculation time. Compared to Nanoscale Molecular Dynamics (NAMD2), it supports a wide range of force fields and has integrated analysis tools that guarantee accurate biomolecular dynamics representation and evaluation. Because of its versatility, OpenMM was used, allowing for customisation for non-standard force fields and integrators via its Python API. For our simulations, the best strategy was to combine the flexibility of OpenMM with the efficiency of GROMACS.

Simulation systems for the specified complexes included the free-protein and the protein in complex with selected inhibitors of the top three best low binding energy ligand-protein complexes post-docking. Each complex was individually solvated within an explicit water box with padding measuring 10 Å, employing the CHARMM36 force field [44] and the TIP3P single-point charge (SPC) water model [45] with periodic boundary conditions (PBCs). To maintain system neutrality, Na^+^ and Cl^−^ ions were introduced. The system underwent an initial energy minimization of 2000 steps followed by a 50 ns production run under the NPT ensemble.

We selected a solution-based system (Figure 1) for our molecular dynamics simulations and inclusion of a lipid bilayer to focus directly on protein–ligand interactions without the added complexity of membrane dynamics. This setup is consistent with the intracellular, cytoplasmic environment where the catalytic domains of VEGFR-2 and c-Met are naturally active, making an aqueous solution a closer approximation than a membrane system. Moreover, in vitro binding and inhibition assays for kinase inhibitors generally take place in solution, which makes our simulation setup more relevant and comparable to the experimental conditions.

Temperature and pressure were regulated using the Nose–Hoover thermostat algorithm and the Martina–Tobias–Klein method. Long-range electrostatic interactions were computed via the Particle-Mesh Ewald (PME) method with a grid spacing of 0.8 Å. Detailed interactions between the ligand and protein were analyzed using the LigPlot+ and analysis packages within the Gromacs and OpenMM packages [28]. Evaluation of the results included examining protein and ligand RMSD, and root mean square fluctuation (RMSF) values relative to a reference structure. To compare the binding properties of the compounds, we performed independent *t*-tests for each interaction distance, using crizotinib and cabozantinib as controls for c-Met and VEGFR-2, respectively. Mean differences were calculated, and statistical significance was set at *p* < 0.05. In addition, 95% confidence intervals were also computed for each comparison.

### 2.12. Visualization and Structural Analysis of Docking/MD Results

Multifacet analysis and visualization of docking results and molecular dynamics (MD) simulation trajectories were performed using an array of computational tools. These included Discovery Studio 3.5 (Biovia) [46,47], LigPlot+ v.2.2, VMD 1.9.4, UCSF Chimera 1.17.1 [24], and PyMOL 2.5. These tools enabled in-depth examination of proteins (VEGFR-2/c-Met) and ligand interactions at both 2D and 3D structural levels, providing atomic-level insights into the binding modes and dynamics of the receptor–inhibitor complexes. MD trajectory visualization further elucidated the conformational changes and stability of the docked poses over the simulation timescale. Additionally, we also utilized our developed molecular visualization platform that enables rapid and interactive display of biomolecules in .pdb format, including our generated VEGFR-2 and c-Met models.

## 3. Results

### 3.1. Structural Modeling of VEGFR-2 Kinase Domain

The kinase domain of VEGFR-2 exhibits a typical bilobed structure characteristic of protein kinases, consisting of an N-terminal lobe and a larger C-terminal lobe (Figure 2A). The N-lobe is primarily composed of five antiparallel β-strands (β1–β5) and one prominent α-helix (αC), while the C-lobe is predominantly α-helical with seven α-helices (αD-αI and αEF) and four short β-strands (β6–β9) (Figure 2A). The ATP-binding site is at the interface between the N- and C-lobes. A glycine-rich loop (residues 841–846) in the N-lobe contains a hydrophobic aromatic residue (F845) (colored in blue licorice (Figure 2A)) positioned near the ATP-binding site. The hinge region, which connects the two lobes (colored in purple ribbon, Figure 2A), plays a crucial role in substrate binding and catalysis. The C-lobe holds the catalytic loop of the conserved TIF (Thr1026-Ile1027-Phe1028) motif essential for catalytic activity. The activation segment (colored in black ribbons, Figure 2A)) that regulates the kinase activity begins with the DFG (D1046-F1047-G1048) motif and ends with the APE (A1073-P1074-E1075) motif. The KEDD motif modulates the tyrosine kinase activity and inhibitor binding is composed of residues K868-E885-D1028-D1046 (Figure 2A). Finally, the kinase domain of VEGFR-2 also features an atypical “split” configuration, with a ~70-residue kinase inserts domain (KID) inserted between the N- and C-terminal lobes of the catalytic tyrosine kinase domain.

### 3.2. Structural Modeling of c-Met Kinase Domain

Similarly, the kinase domain of c-Met (Figure 3) exhibits a typical bilobed structure characteristic of protein kinases, consisting of an N-terminal lobe and a larger C-terminal lobe connected by the hinge region (colored in yellow ribbon) [20]. The N-lobe is mainly constituted of β-strands and features a notable α-helix, whereas the C-lobe is primarily composed of α-helices, interspersed with several β-strands. The ATP-binding site is at the interface between the N- and C-lobes separated the hinge region. This site plays a crucial role in the catalytic activity of the kinase domain. Like other kinases, c-Met contains a conserved DFG (Asp1222-Phe1223-Gly1224) motif that plays a crucial role in kinase activation and inhibitor binding. The catalytic pocket contains distinguished features, which include the flexible activation loop (spans from approximately residue 1230 to 1255 colored in orange) that changes conformationally to regulate access to the ATP-binding site. The catalytic loop colored in blue contains conserved residues, including K1110, E1127, D1222, G1224, and the catalytic tyrosine residues (Y1234 and Y1235, in cartoon presentation), and the HRD conserved motif (His-1248, Arg-1249, Asp-1250) believed to participate in the catalytic activity [20].

### 3.3. Hierarchical Ranking of Potential VEGFR-2 and c-Met Kinase Inhibitors

A molecular docking and screening approach (Figure 1) was conducted using a library of 2016 FDA-approved drugs against two molecular targets that is c-Met and VEGFR-2. Our molecular docking and dynamics simulations focused on the kinase domains of VEGFR-2 and c-Met, as these regions contain the critical catalytic residues responsible for ATP binding and phosphorylation, which are the primary targets for small molecule inhibitors. The top-ranking compounds were selected (Table 1) based on three key parameters: binding affinity, distance to catalytic residues, and log odds score (Table 2). As controls, crizotinib for c-Met and cabozantinib for VEGFR-2 were included in the selection process. Despite their diversity, one potential commonality among several of these drugs is the presence nitrogen containing rings found in cabozantinib, crizotinib, telmisartan, triamterene, dihydroergotamine, and fludarabine phosphate.

**Figure 4:**
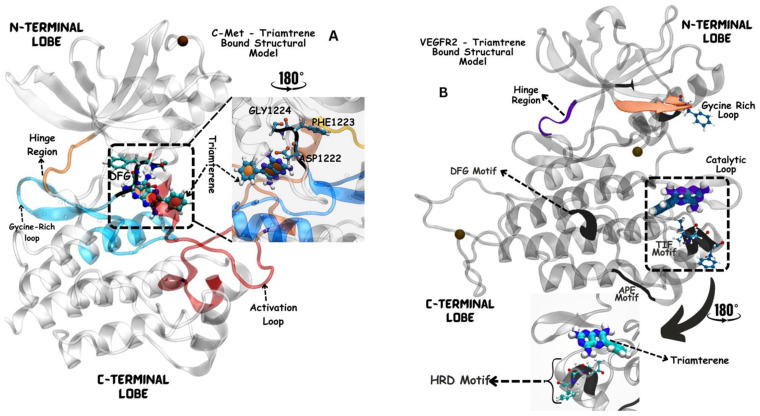
Binding mode of triamterene in the kinase domains of c-Met (**A**) and VEGFR-2 (**B**). (**A**) Triamterene binding to c-Met kinase domain. The inhibitor (shown in licorice representation) occupies the ATP-binding site in the cleft between the N- and C-lobes, functioning as a competitive inhibitor. Triamterene is observed close to the DFG motif and highly conserved residues in the catalytic loop (Gly1224, Phe1223, and Asp1222 (**A**)). Triamterene binding to VEGFR-2 kinase domain (**B**). The inhibitor is positioned near the canonical binding site within the ATP-binding pocket. Notably, triamterene interacts with residues in the activation loop, whose orientation is critical for kinase activity and inhibitor binding.

Moreover, the aromatic rings identified in cabozantinib, crizotinib, entacapone, eltrombopag, telmisartan, mizolastine, and salsalate are hypothesized to engage in π-π interactions, which occur between the π-electron clouds of aromatic rings. The structural insights reveal the molecular basis for triamterene’s inhibitory action on c-Met (Figure 4A) and VEGFR-2 (Figure 4B) kinases, highlighting its potential as a dual-target therapeutic agent. The distinct binding modes in each kinase underscore the importance of structural analysis in understanding ligand–protein interactions and guiding structure-based drug design efforts. The screening identified several compounds that exhibited strong binding properties to both kinases as a function of the binding affinity, distance to the binding site and the odds score (Table 2). It is important to note that while these compounds showed promising results as single inhibitors for each target, their potential for dual inhibition was inferred from their performance across both proteins.

**Figure 5:**
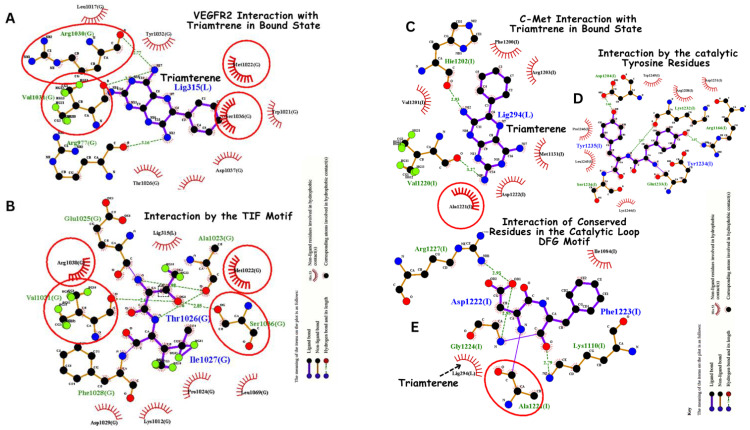
Comparative analysis of triamterene binding to VEGFR-2 and c-Met kinase domains. Triamterene binding to VEGFR-2 kinase domain. (**A**) indicates hydrogen bond interactions with Arg1030 (via the oxygen group), Val1031, and Arg977. Importantly, triamterene interacts with the TIF motif (**B**) (Thr1024, Ile1025, Phe1026). (**C**). Interaction of triamterene with c-Met, hydrogen bonds (in dashed lines) with His1202 and Val1220 via the oxygen group, and hydrophobic interactions (in red line) with Ala1221, Asp1222, Met1131, Arg1203, and Phe1200. Notably, triamterene is close to the DFG motif (**E**). Asp1222, Phe1223, Gly1224) and the highly conserved Tyrosine residues (**D**) in the catalytic loop.

Triamterene bonding interaction to both VEGFR-2 and c-Met is analyzed as shown in Figure 5. Triamterene exhibits a significant binding affinity of −8 kcal/mol and −8.0 kcal/mol on both VEGFR-2 and c-Met in computational screening. Triamterene binds to VEGFR-2 through hydrogen bonding with Arg1030 (via the oxygen group), Val1031 and Arg977 with more specific interaction with TIF motif. These key moieties allow for favorable interactions with the binding pocket of both VEFGR-2 as shown in Figure 5. On the other hand, triamterene interacts with c-Met through hydrogen bonding with His1202 and Val1220 via the oxygen group, and through hydrophobic interactions with Ala1221, Asp1222, Met1131, Arg1203 and Phe1200 (Figure 5). As an FDA-approved drug primarily as a potassium-sparing diuretic, triamterene has a well-documented safety profile in humans, potentially facilitating its repurposing for cancer therapy. Triamterene’s potential as an anticancer agent, especially as a dual inhibitor, provides an inventive approach to medication repurposing, even if its main usage is in the treatment of edema and hypertension.

### 3.4. Virtual Screening: Top 11 Selected Ligands for c-Met and VEGFR-2

The virtual screening process identified and ranked the top 11 ligands for c-Met and VEGFR based on their binding affinity, proximity to the binding pocket, and log-odds score (Table 2). The selected ligands included cabozantinib, crizotinib, dihydroergotamine, eltrombopag, entacapone, fludarabine phosphate, mizolastine, oxandrolone, salsalate, telmisartan, and triamterene (Figure 3 and Figure 4). Below is a detailed analysis of their rankings, including their formulas, molecular weights, and 2D/3D presentations (Table 1). These compounds demonstrated the most favorable predicted interactions with the target protein binding sites. It was found that these two receptors have distinct single inhibitors: entacapone for c-Met and telmisartan for VEGFR-2. However, one drug, triamterene, was identified as a common inhibitor for both receptors in cancer.

### 3.5. Molecular Dynamics-Driven Identification of lead Drug Interactions with c-Met and VEGFR-2 Catalytic Domains

**Figure 6:**
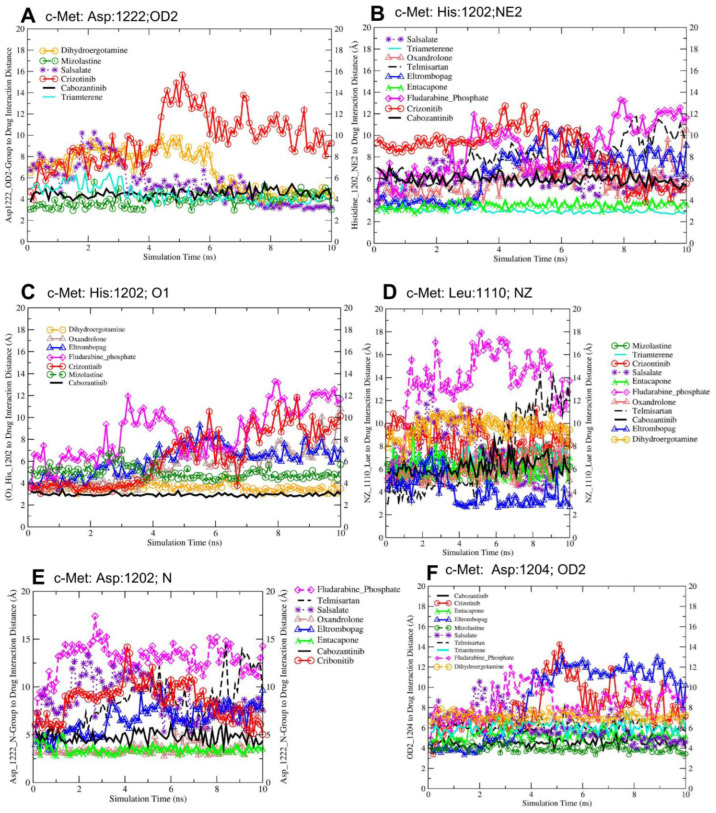
Molecular Dynamics Simulation of Drug–Residue Interactions in c-Met Target Protein. This figure illustrates the interactions between screened drug candidates and key residues of the c-Met protein, observed during the final 10 ns of the molecular dynamic simulation production run. The distances (in Ångströms) between the drugs and specific residue atoms are presented, providing a dynamic overview of the stability and nature of these interactions over time. Each drug is color-coded for clarity, enabling easy comparison of binding patterns and interaction strengths. These findings contribute to understanding the binding efficiency and specificity of the screened compounds with the c-Met target. (**A–F**) show the molecular interactions of the selected drugs with active residues (amino acids) of c-Met protein at the atomic level.

Molecular dynamics simulations (50 ns but this paper includes the last 10 ns of the production run) revealed significant interactions between c-Met’s catalytic domains and selected FDA-approved lead drugs, involving key residues Leu1110, Asp1204, His1202, and Asp1222 (Figure 6). Entacapone exhibited specific c-Met inhibition, while triamterene demonstrated dual inhibitory potential for both c-Met and VEGFR-2. The protein–drug interactions, particularly with Asp1204 of c-Met, were visualized for various FDA-approved drugs. Similarly, VEGFR-2 catalytic domains showed notable interactions with screened drugs, primarily involving residues Lys868, Asp1028, and Asp1046. These findings elucidate potential drug repurposing opportunities for c-Met and VEGFR-2 inhibition. The trajectory visualization files have been provided through the following link (https://github.com/lutimbastuart/MolecularDynamics-cMet-VEGFR2 (accessed on 1 August 2024)).

The interaction with Asp1222 (OD2) indicates that mizolastine, cabozantinib, and triamterene form the strongest hydrogen bonding interactions with the Asp1222 residue, as evidenced by their shorter interaction distances compared to the other drugs (Table 3). Triamterene, the selected dual inhibitor, showed significantly lower distances across all measured interactions compared to crizotinib (*p* < 0.0001), with mean differences ranging from −1.61 Å to −5.38 Å. Telmisartan, the selected single inhibitor, also demonstrated significantly lower distances for most interactions (*p* < 0.0001), with mean differences between −0.58 Å and −3.58 Å, except for the Asp1202(N) interaction where the difference was not significant. The longer distances for dihydroergotamine, salsalate, fludarabine phosphate, and crizotinib suggest weaker or more transient interactions at this site delineated in Figure 6A. The average distances between His1202 (NE2) and the interacting drugs indicates that cabozantinib and mizolastine form the strongest interactions with the His1202 residue, while crizotinib, eltrombopag, and fludarabine phosphate have the weakest interactions at this site shown in Figure 6B. The interaction with His1202 at the O1 group indicates that triamterene and entacapone show the strongest interactions with the His1202 (O1) atom, while fludarabine phosphate has the weakest interaction at this site, as presented in Figure 6C.

The average distances between Leu1110 (NZ) indicates that eltrombopag forms the strongest interaction with the Leu1110 (NZ) atom, while fludarabine phosphate has the weakest interaction at this site (Figure 6D). The average distances between Asp1202 (N) indicates that entacapone and oxandrolone show the strongest interactions with the Asp1202 (N) atom, while fludarabine phosphate has the weakest interaction at this site (Figure 6E).

**Figure 7:**
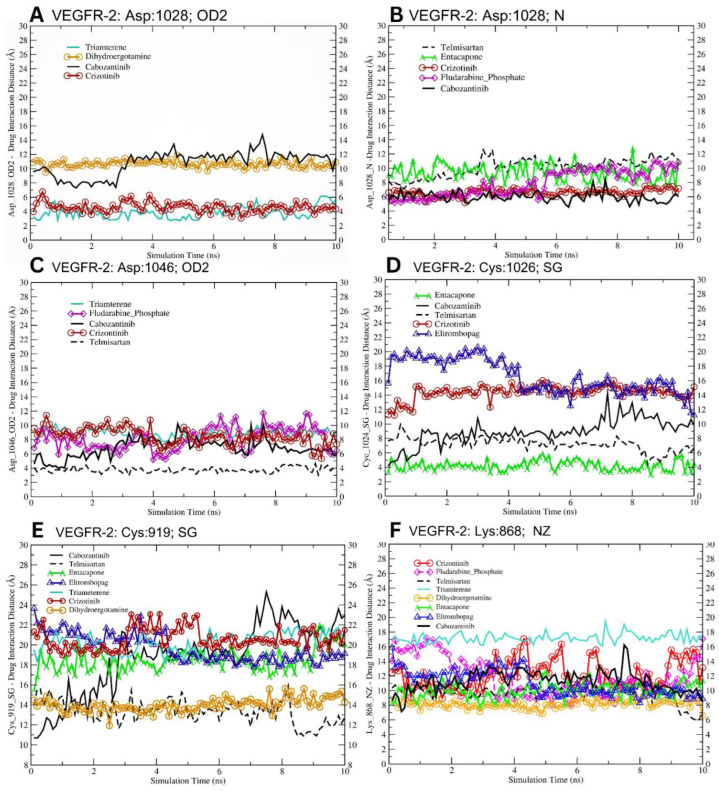
Molecular dynamics simulation of drug–residue interactions in VEGFR-2 target protein. This figure depicts the interactions between screened drug candidates and key residues of the VEGFR-2 protein, captured during the final 10 ns of the molecular dynamic simulation production run. The distances (in Ångströms) between the drugs and specific residue atoms are plotted, providing a detailed view of the dynamic behavior and stability of the interactions over time. Each drug is represented by a distinct color for clarity, highlighting variations in binding patterns and interaction strengths. This analysis offers insights into the potential binding efficacy and specificity of the screened compounds with VEGFR-2. (**A–F**) show the molecular interactions of the selected drugs with active residues (amino acids) of VEGFR-2 protein at the atomic level.

Our molecular docking studies revealed diverse interaction patterns between the VEGFR-2 catalytic domain and several potential inhibitors. The analysis focused on key residues within the binding pocket, including Cys919, Cys1024, Lys868, Asp1028, and Asp1046 (Table 4 and Figure 7). The thiol group of Cys919 exhibited variable interaction distances with the tested compounds (Table 4 and Figure 7E). Notably, telmisartan and dihydroergotamine showed considerable interactions (13.35 Å and 13.99 Å, respectively) compared to other compounds such as cabozantinib (18.76 Å) and crizotinib (20.59 Å).

The ε-amino group of Lys868 showed a range of interaction distances (Figure 7F). Dihydroergotamine exhibited the closest interaction (8.17 Å), while telmisartan and entacapone also showed relatively close interactions (9.64 Å and 10.02 Å, respectively, Figure 7F). Cabozantinib and crizotinib displayed moderate interaction distances of 11.32 Å and 12.14 Å, respectively. Asp1028, through its OD2 group, showed strong interactions with triamterene (3.85 Å) and crizotinib (4.59 Å). Other compounds such as cabozantinib and dihydroergotamine exhibited longer interaction distances (10.78 Å and 10.66 Å, respectively, Figure 7A). These results indicate that triamterene binds specifically to the Asp1028 residue within the VEGFR-2 catalytic site, rather than competing directly in the ATP-binding pocket.

This interaction suggests a potential allosteric inhibition mechanism, where triamterene disrupts the proton transfer required for phosphorylation and kinase activation. Binding distance and affinity measurements confirm triamterene’s stable association with Asp1028 and its high specificity, setting it apart from ATP-competitive inhibitors like cabozantinib.

Triamterene, the selected dual inhibitor, showed significantly different interactions compared to cabozantinib for all measured amino acids (*p* < 0.0001), with notably longer distances for Cys919 (+1.51 Å), Lys868 (+5.88 Å), and Asp1046 (+2.08 Å), but a shorter distance for Asp1028 (−6.94 Å). Entacapone, the selected single inhibitor, exhibited significantly shorter distances for Cys1024 (−4.72 Å) and Lys868 (−1.30 Å) (*p* < 0.0001), while its interaction with Cys919 was not significantly different from cabozantinib (*p* > 0.05).

**Figure 8:**
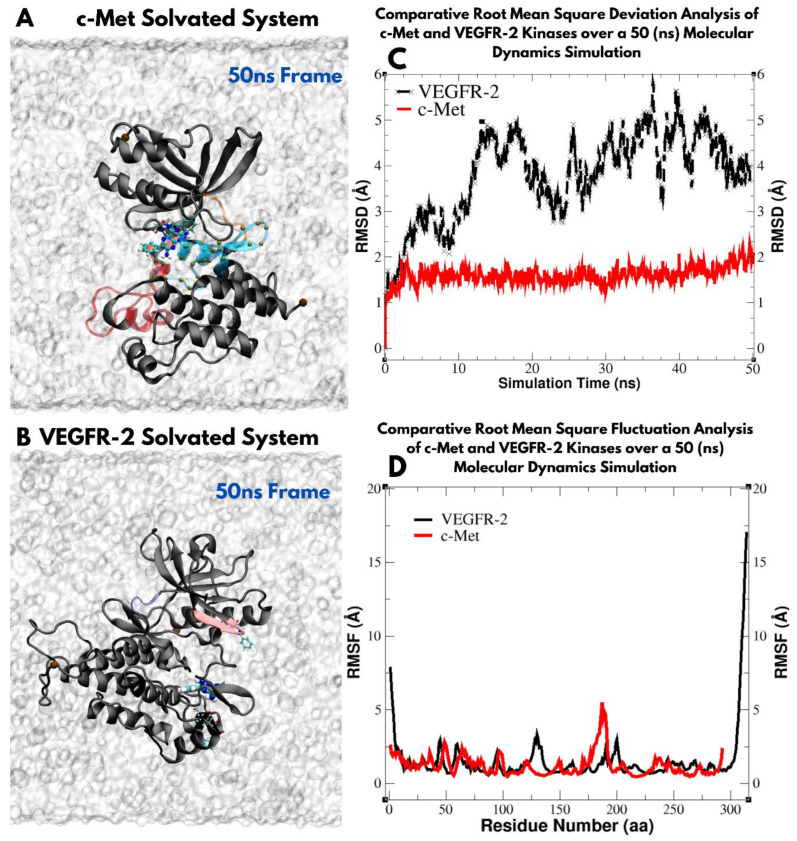
Molecular dynamics simulation analysis of c-Met and VEGFR-2 kinases over a 50 ns period. Panels (**A**,**B**) depict the solvated systems of c-Met and VEGFR-2, respectively, with each protein shown as black cartoon representations to highlight structural features, while water molecules are visualized as gray surface representations. Panel (**C**) presents the Root Mean Square Deviation (RMSD) of c-Met (red) and VEGFR-2 (black), reflecting their structural stability during the simulation. Panel (**D**) displays the Root Mean Square Fluctuation (RMSF) for both proteins, with c-Met in red and VEGFR-2 in black, indicating flexibility across individual residues over the simulation timeframe.

Asp1046 demonstrated varied interaction patterns. Telmisartan showed the closest interaction (3.78 Å), followed by cabozantinib (6.81 Å). Fludarabine phosphate, crizotinib, and triamterene all exhibited moderate interaction distances ranging from 8.15 Å to 8.89 Å. Cabozantinib, a known potent VEGFR-2 inhibitor, showed consistent moderate to strong interactions across all analyzed residues. This multi-point interaction pattern may contribute to its high efficacy. Telmisartan demonstrated notably close interactions with both Asp1046 (3.78 Å) and Lys868 (9.64 Å), suggesting a potential for strong binding within the catalytic domain (Figure 7C,F, respectively). Entacapone showed the closest interaction with Cys1024 (4.22 Å) among all tested compounds, indicating a possible key interaction point for VEGFR-2. These results provide interaction insights into the binding modes of various selected lead compounds within the VEGFR-2 catalytic domain, highlighting potential structural interactions that could guide future drug design efforts targeting this important receptor tyrosine kinase. Further, we expanded our analysis to evaluate the structural stability and flexibility of VEGFR-2 and c-Met protein kinases over a 50 ns (nanosecond) timeframe in a solvated environment. As shown in Figure 8C, the Root Mean Square Deviation (RMSD) is stable for c-Met within 1–2 Å, while fluctuating in VEGFR-2 within 1–5 Å indicating conformational change over the 50 ns range. The Root Mean Square Fluctuation (RMSF) is relatively similar for both VEGFR-2 and c-Met proteins showing similar flexibility patterns of individual residues in the two proteins (Figure 8D). 

## 4. Discussion

The landscape of cancer therapeutics has been dominated by single-target inhibitors, focusing on specific molecular pathways such as VEGFR-2 and c-Met signaling [8]. VEGFR-2 [48,49], a key player in angiogenesis, and c-Met [17,20], crucial for cell proliferation and survival, are often overexpressed or aberrantly activated in different types of cancer including prostate, lung and liver cancers. The current study employed the dual inhibition of VEGFR-2 and c-Met receptors as a promising therapeutic strategy over mono-targeted therapy. The study revealed that telmisartan and entacapone are potent and selective inhibitors of VEGFR-2 and c-Met, respectively, while triamterene emerges as a promising dual inhibitor as evidenced by robust molecular docking and dynamic simulation analyses (Figure 1).

Telmisartan, an inhibitor of angiotensin II receptor used primarily to treat hypertension, has shown selective inhibition of VEGFR-2 required for angiogenesis involved in cancer growth and metastasis [49]. Anti-VEGFR-2 therapeutics such as bevacizumab have been reported to disrupt tumour angiogenesis with significant clinical efficacy [5,50]. Telmisartan’s ability to selectively inhibit VEGFR-2 suggests its potential as a therapeutic option targeting angiogenesis during tumour development. Entacapone, a COMT (catechol-O-methyltransferase) inhibitor used in Parkinson’s disease management, showed a potent and selective inhibition of c-Met receptor in the current study [51]. The c-Met-mediated signaling pathway is involved in cell proliferation, survival, migration and metastasis of cancer cells [17]. Various studies have shown that c-Met is dysregulated in different cancers including gastric, lung and liver cancers [52]. Selective inhibition of c-Met by entacapone provides a promising therapeutic drug against these c-Met driven malignancies [51].

For VEGFR-2, drugs like cabozantinib, sunitinib and sorafenib have shown efficacy in certain cancer types such as colorectal cancer (CRC) [53]. Similarly, c-Met inhibitors such as crizotinib and cabozantinib have demonstrated promising binding strength in the inhibition of c-Met and VEGFR-2 receptors as shown in Table 2, consistent with the existing literature [14,54]. However, the limitations of these monotherapies have become increasingly apparent. Single-target inhibitors, while effective initially, often lead to the activation of compensatory pathways or the selection of resistant cell populations. Dual inhibitors, by targeting both VEGFR-2 and c-Met simultaneously, offer the potential to circumvent these resistance mechanisms and provide more durable responses.

Notably, in the current study, we identified triamterene, a potassium-sparing diuretic, as a dual inhibitor with specific binding affinity to VEGFR-2 and c-Met receptors. In comparison to cabozantinib in VEGFR-2, which is known to bind directly within the ATP-binding pocket, triamterene’s orientation at Asp1028 highlights a distinct binding dynamic. This observation supports the hypothesis that triamterene may modulate VEGFR-2 activity through a novel, non-competitive pathway, potentially altering the kinase’s functionality Via an allosteric interaction rather than direct ATP competition. Triamterene as a dual inhibitor of both protein targets provide a strong therapeutic potential compared to single-receptor inhibitors to inhibit angiogenesis and invasiveness of cancer cells. The distinct binding patterns of triamterene and entacapone, compared to cabozantinib, suggest potential mechanisms for their inhibitory effects. Triamterene’s dual inhibition may be attributed to its significantly altered interactions across multiple amino acids, particularly the substantial changes in Lys868 and Asp1028 distances. Entacapone’s potent single inhibition could be related to its significantly closer interactions with Cys1024 and Lys868, potentially indicating a more focused binding mechanism.

Dual inhibition strategies have shown significant inhibition of cancer progression in clinical settings; for instance, foretinib [10] and cabozantinib [6] have been previously reported to suppress tumour growth and metastasis in medullary thyroid cancer and renal cell carcinoma. However, triamterene has a significant binding affinity to both VEGFR-2 and c-Met and has the potential for more robust suppression of tumour growth and metastasis. These interactions are crucial for understanding the mechanism of action of dual inhibitors and their potential downstream effects. For c-Met, key interactions were observed with residues such as Asp1204, His1202, and Asp1222 (Figure 4). The binding mode of triamterene to c-Met exhibits characteristics of a type 1 inhibition, occupying the ATP-binding site in proximity to the DFG motif as observed in Figure 4. This interaction induces a conformational change in the DFG motif, with the aspartate residue engaging the triamterene substrate. The tryptophan residue’s orientation away from triamterene suggests that the inhibitor occupies the space typically reserved for ATP in the active c-Met conformation (Figure 4). These observations collectively indicate that triamterene functions as a competitive inhibitor of c-Met by directly competing with ATP for the binding site. These residues are known to play vital roles in the kinase activity of c-Met [55,56].

The analysis of distance metrics between potential inhibitors and key residues in the catalytic domains of VEGFR-2 and c-Met provides valuable insights into the nature and strength of these interactions. This approach aligns with previous studies that have emphasized the importance of specific residues in the binding pockets of these kinases. For VEGFR-2, our analysis focused on critical residues such as Cys919, Cys1024, Lys868, Asp1028, and Asp1046. The varying interaction distances observed with these residues across different compounds reflect the diverse binding modes of potential inhibitors. For instance, the closer interactions of telmisartan and dihydroergotamine with Cys919 (13.35 Å and 13.99 Å, respectively) compared to cabozantinib (18.76 Å) suggest potential differences in binding affinity and mechanism. The interaction patterns observed with Lys868 are particularly noteworthy, given its known importance in VEGFR-2 inhibition. The close interaction of dihydroergotamine (8.17 Å) with this residue suggests a potentially strong binding mode, which could be explored further in drug design studies.

The binding affinity of triamterene to the kinase domains of c-Met and VEGFR-2 showed negligible differences between the isolated kinase domains and the full-length protein structures, highlighting their structural equivalence. The RMSD values further confirmed this similarity, with 0.82 Å for c-Met and 0.76 Å for VEGFR-2 when comparing the full-length structures to the kinase domains alone. Additionally, the binding modes of triamterene and the control inhibitors were consistent, with RMSDs of 1.24 Å (c-Met: triamterene vs. crizotinib) and 1.18 Å (VEGFR-2: triamterene vs. cabozantinib).

For c-Met, the analysis of interactions with residues like Asp1222, His1202, Leu1110, and Asp1202 revealed varying strengths of interactions across different compounds. The strong hydrogen bonding interactions of mizolastine, cabozantinib, and triamterene with Asp1222, as indicated by shorter interaction distances, align with previous findings on the importance of this residue in c-Met inhibition [20,21]. The observed interaction patterns of cabozantinib with both VEGFR-2 and c-Met residues corroborate its known dual inhibitory activity. Its consistent moderate to strong interactions across multiple residues in both kinases provides a molecular basis for its efficacy as a dual inhibitor. These distance metric analyses not only confirm existing knowledge about key interactions in VEGFR-2 and c-Met inhibition but also highlight potential new interaction patterns.

For instance, the strong interaction of triamterene and telmisartan with Asp1046 in VEGFR-2 (3.78 Å) suggests a possible mechanism for its reported anti-angiogenic effects. The varying interaction strengths observed across different compounds provide valuable structure-activity relationship insights. These findings can guide future medicinal chemistry efforts in optimizing lead compounds for improved binding to VEGFR-2 and c-Met, potentially leading to more effective dual inhibitors.

The binding affinity values observed in this study provide valuable insights into the potential therapeutic efficacy of inhibitors targeting VEGFR2 and c-Met. Stronger binding, indicated by more negative values, correlates with the potential for more potent inhibition of these receptors in clinical settings, which play key roles in cancer-related processes such as angiogenesis and cell proliferation. For instance, the single inhibitors entacapone and telmisartan demonstrated significant binding affinities of −9.7 kcal/mol and −10.2 kcal/mol, respectively, for their respective targets. Notably, triamterene, a dual inhibitor, showed favourable binding affinities of −8.9 kcal/mol for c-Met and −9.1 kcal/mol for VEGFR-2, suggesting its potential to disrupt multiple cancer-related pathways. While these binding affinity values are promising indicators of biological activity and a promising start point, further experimental validation is required to establish a direct correlation with therapeutic efficacy in clinical settings.

While the findings from the current study are promising, it is important to acknowledge certain limitations and assumptions inherent in computational models. Our focus on the kinase domains, while well-established in structure-based drug design, does not account for potential influences from other protein domains. The use of crystal structures provides a static view that may not fully capture the proteins’ dynamic nature in physiological conditions. Additionally, the timescale of our molecular dynamics simulations (50 ns) and the limitations of scoring functions and solvent models may not encompass all aspects of protein–ligand interactions. Also, computational tools do not include complex biological environments overlooking drug pharmacokinetics, pharmacodynamics and tumour microenvironment factors. Despite these limitations, our approach provides a solid foundation for identifying potential dual inhibitors, which can be further validated through experimental studies.

Further research is also needed to confirm and translate these preclinical analyses into clinical applications. Although the mechanism of action of triamterene as a dual VEGFR-2 and c-Met inhibitor has not been thoroughly investigated, prior research has also demonstrated its potential anticancer characteristics. Repurposing triamterene, a potassium-sparing diuretic, for targeting VEGFR-2 and c-MET presents potential therapeutic advantages [57]. However, it also raises concerns regarding off-target and side effects that warrant consideration [58]. By blocking renal tubules’ epithelial sodium channels (ENaC), triamterene is known to lower potassium excretion [59]. Although this activity helps control heart failure and hypertension, it may make cancer patients’ hyperkalemia worse, especially if they have impaired renal function. Triamterene’s suppression of VEGFR2 and c-MET may also have unforeseen metabolic and cardiovascular consequences [58,60].

VEGFR2 suppression may also be a factor in hypertension, thrombotic events, and delayed wound healing since it is associated with endothelial dysfunction and reduced angiogenesis [60,61]. However, c-Met inhibition may interfere with tissue regeneration and cellular repair, raising the risk of hepatic and gastrointestinal toxicity in susceptible patients [60]. Hence, in vitro therapeutic efficacy and toxicity of telmisartan, entacapone and triamterene are required to accurately predict their clinical outcomes. Second, molecular mechanistic studies of these inhibitors are essential to optimize therapeutic strategies. In addition, combination of these drugs with existing chemotherapies or targeted therapies might have a potential to enhance overall treatment efficacy.

## 5. Conclusions

The identification of a potential dual inhibitor of both VEGFR-2 and c-Met, triamterene, presents a promising strategy. This finding presents an opportunity to improve the effectiveness of anticancer drugs associated with resistance in clinical settings. The molecular mechanisms and interactions revealed through this study provide a strong foundation for understanding how these dual inhibitors exert their inhibitory effects. With respect to VEGFR-2, key interactions were observed with residues such as Lys868, Asp1028, and Asp1046. Significant interactions in c-Met were observed with Asp1204, His1202 and Asp1222. We thus identified entacapone as a potent and selective inhibitor for c-Met, and telmisartan as a selective inhibitor for VEGFR-2. While the effectiveness of dual inhibition is significant, it is crucial to approach this strategy with a balanced perspective. By simultaneously inhibiting both VEGFR-2 and c-Met, triamterene may offer a strategy to overcome the resistance mechanisms associated with single-target therapies. Notably, triamterene emerged as a promising dual inhibitor, demonstrating significant binding affinity to both VEGFR-2 and c-Met proteins. The potential for off-target effects and the emergence of novel resistance mechanisms must be carefully considered and studied. The identification of triamterene as a dual inhibitor justifies its estimation for targeting multiple several signaling pathways not only those involved in cancer progression, metastasis, but also poor prognosis.

Our list of screened compounds are FDA-approved drugs that have well-established safety profiles, having undergone extensive human testing. This reduces the risk of unexpected adverse effects during clinical trials. The pharmacokinetics and pharmacodynamics of these drugs are already well-understood, facilitating dose calculations and predicting potential side effects. However, comprehensive preclinical investigations are necessary to analyze the cytotoxicity, selectivity and functional assays of the drugs in cancer cell lines expressing these receptors. The efficacy of these compounds, particularly triamterene, should also be tested in relevant cancer models such as 3D cancer spheroids and animal models. In addition, subsequent clinical trials will be crucial to assess the safety and efficacy of triamterene in human patients, especially in patients showing resistance to current chemotherapies. Future research should also explore potential synergistic effects of these selected drugs with existing chemotherapies. Combining these approaches with computational tools underscores the utility of this pipeline in accelerating the identification and assessment of novel therapeutic drugs for precision medicine.

## Figures and Tables

**Table 1:** Virtual screening results for top-ranked drugs targeting c-Met and VEGFR-2. This table showcases the virtual screening results for the top-ranked and selected drugs identified as dual-target inhibitors of c-Met and VEGFR-2. For each drug, the table includes the PubChem CID, molecular formula, molecular weight (in g/mol), canonical SMILES (Simplified Molecular Input Line Entry System) representation, and 2D structural depiction. The molecular formula and weight provide essential information about the drug’s composition and size, while the SMILES and 2D structures enable visualization of the chemical framework. These details form the basis for understanding the structural properties influencing the drugs’ binding efficacy and dual-target potential.

Name and PubChem CID	Molecular Formular	Molecular Weight {g/mol}	Canonical SMILES	2D Structure
Cabozantinib	C_28_H_24_FN_3_O_5_	501.5	COC1=CC2=C(C=CN=C2C=C1OC)OC3=CC=C(C=C3)NC(=O)C4(CC4)C(=O)NC5=CC=C(C=C5)F	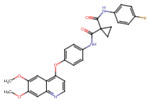
Crizotinib	C_21_H_22_Cl_2_FN_5_O	450.3	CC(C1=C(C=CC(=C1Cl)F)Cl)OC2=C(N=CC(=C2)C3=CN(N=C3)C4CCNCC4)N	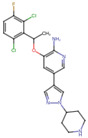
Entacapone	C_14_H_15_N_3_O_5_	305.29	CCN(CC)C(=O)C(=CC1=CC(=C(C(=C1)O)O)[N+](=O)[O-])C#N	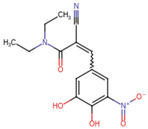
Eltrombopag	C_25_H_22_N_4_O_4_	442.5	CC1=NN(c2ccc(C)c(C)c2)C(=O)/C1=N\Nc1cccc(c2cccc(C(=O)O)c2)c1O	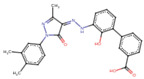
Telmisartan	C_33_H_30_N_4_O_2_	514.6	CCCC1=NC2=C(N1CC3=CC=C(C=C3)C4=CC=CC=C4C(=O)O)C=C(C=C2C)C5=NC6=CC=CC=C6N5C	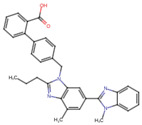
Triamterene	C_12_H_11_N_7_	253.26	C1=CC=C(C=C1)C2=NC3=C(N=C(N=C3N=C2N)N)N	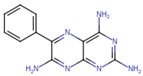
Dihydroergotamine	C_33_H_37_N_5_O_5_	583.7	CC1(C(=O)N2C(C(=O)N3CCCC3C2(O1)O)CC4=CC=CC=C4)NC(=O)C5CC6C(CC7=CNC8=CC=CC6=C78)N(C5)C	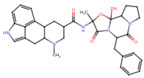
Fludarabine Phosphate	C_10_H_13_FN_5_O_7_P	365.21	C1=NC2=C(N=C(N=C2N1C3C(C(C(O3)COP(=O(O)O)O)O)F)N	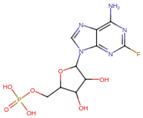
Mizolastine	C_24_H_25_FN_6_O	432.5	CN(C1CCN(CC1)C2=NC3=CC=CC=C3N2CC4=CC=C(C=C4)F)C5=NC=CC(=O)N5	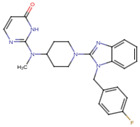
Oxandrolone	C_19_H_30_O_3_	306.4	CC12CCC3C(C1CCC2(C)O)CCC4C3(COC(=O)C4)C	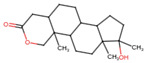
Salsalate	C_14_H_10_O_5_	258.2	C1=CC=C(C(=C1)C(=O)OC2=CC=CC=C2C(=O)O)O	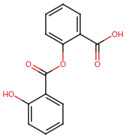

**Table 2:** Comparative Analysis of Potential Dual c-Met and VEGFR-2 Inhibitors. This table provides a comparative analysis of candidate drugs identified as potential dual inhibitors of the c-Met and VEGFR-2 targets. The data include binding affinities (in kcal/mol) for each target, proximity to the catalytic site (in Ångströms, Å), and log-odds (LOD) scores for both c-Met and VEGFR-2. Binding affinities indicate the strength of interactions, with more negative values reflecting stronger binding. Proximity values denote the spatial closeness of the drugs to the catalytic site, which is critical for inhibitory activity. Log-odds scores provide additional metrics for evaluating the likelihood of effective binding and inhibition. This comprehensive analysis highlights the relative potential of each candidate drug for dual-target inhibition.

No:	Candidate Drugs	Binding Affinity in c-Met Target (kcal/mol)	Binding Affinity in VEGFR-2 Target (kcal/mol)	Proximity to the Catalytic Site in c-Met (Å)	Proximity to the Catalytic Site in VEGFR-2 (Å)	Log Odds (LOD) Score in c-Met	Log Odds (LOD) Score in VEGFR-2
1.	Cabozantinib	−7.7	−8.2	3.1	2.9	−0.55	−0.52
2.	Crizotinib	−7.9	−7.5	2.4	3.1	−0.64	−0.60
3.	Telmisartan	−7.3	−7.8	3.2	2.8	−15.3	−18.7
4.	Triamterene	−8.0	−8.3	2.5	2.7	−0.50	−0.6
5.	Dihydroergotamine	−7.6	−7.4	2.9	3.0	−12.5	−11.8
6.	Oxandrolone	−7.7	−7.1	3.3	3.5	−5.7	−8.2
7.	Mizolastine	−7.0	−7.3	3.1	2.7	−8.2	−10.4
8.	Fludarabine Phosphate	−7.4	−7.0	2.3	2.6	−6.64	−9.62
9.	Eltrombopag	−7.8	−8.0	2.7	2.5	−22.3	−25.1
10.	Entacapone	−7.5	−7.2	2.2	2.4	−19.0	−17.5
11.	Salsalate	−6.8	−6.6	3.4	3.6	−37.18	−40.5

**Table 3:** Average interaction distances of lead drugs at functional sites with key residues of c-Met target protein. This table summarizes the average interaction distances (in Ångströms, Å) between lead drug compounds and critical amino acid residues in the active site of the c-Met protein, as determined through molecular docking simulations. Average interaction distances of lead drugs at functional sites with key residues of c-Met target protein as illustrated in Figure 3.

Lead Drug	Asp1222 (OD2) Å	His1202 (NE2) Å	His1202 (O1) Å	Leu1110(NZ) Å	Asp1202(N) Å	Asp1204 (OD2) Å
Cabozantinib	4.54 ± 0.04 *** (−3.55)	4.54 ± 0.05 *** (−5.15)	5.95 ± 0.01 *** (−2.48)	6.19 ± 0.07 *** (−2.41)	4.77 ± 0.06 *** (−3.80)	4.54 ± 0.14 *** (−5.15)
Crizotinib	8.09 ± 0.19	9.69 ± 0.22	8.43 ± 0.25	8.60 ± 0.13	8.57 ± 0.19	9.69 ± 0.31
Dihydroergotamine	6.75 ± 0.21 *** (−1.34)	6.75 ± 0.04 *** (−2.94)	-	-	-	6.75 ± 0.31 *** (−2.94)
Eltrombopag	-	8.75 ± 0.22 *** (−0.94)	6.65 ± 0.13 *** (−1.78)	3.86 ± 0.12 *** (−4.74)	6.58 ± 0.15 *** (−1.99)	8.75 ± 0.05 *** (−0.94)
Entacapone	-	5.30 ± 0.03 *** (−4.39)	3.51 ± 0.22 *** (−4.92)	6.71 ± 0.08 *** (−1.89)	3.47 ± 0.04 *** (−5.10)	5.30 ± 0.62 *** (−4.39)
Fludarabine Phosphate	-	8.49 ± 0.14 *** (−1.20)	13.18 ± 0.23 *** (4.75)	13.59 ± 0.30 *** (4.99)	12.88 ± 0.17 *** (4.31)	8.49 ± 0.29 *** (−1.20)
Mizolastine	3.89 ± 0.06 *** (−4.20)	4.02 ± 0.06 *** (−5.67)	-	6.32 ± 0.08 *** (−2.28)	-	4.02 ± 0.41 *** (−5.67)
Oxandrolone	-	-	5.84 ± 0.16 *** (−2.59)	5.83 ± 0.10 *** (−2.77)	3.67 ± 0.11 *** (−4.90)	-
Salsalate	6.32 ± 0.32 *** (−1.77)	6.34 ± 0.21 *** (−3.35)	-	7.33 ± 0.24 *** (−1.27)	8.72 ± 0.18 (0.15) ^NS^	6.34 ± 0.43 *** (−3.35)
Telmisartan	-	6.11 ± 0.23 *** (−3.58)	7.85 ± 0.31 *** (−0.58)	7.21 ± 0.22 *** (−1.39)	8.39 ± 0.51 (−0.18) ^NS^	6.11 ± 0.33 *** (−3.58)
Triamterene	4.54 ± 0.24 *** (−3.55)	6.07 ± 0.17 *** (−3.62)	3.05 ± 0.34 *** (−5.38)	6.99 ± 0.12 *** (−1.61)	-	6.07 ± 0.23 *** (−3.62)

Note: A dash (-) indicates no interaction data available for the specific drug and residue pair. Values in parentheses represent the mean difference compared to crizotinib. *** indicates *p* < 0.0001 (highly significant difference from crizotinib). ^NS^ (not significant) indicates not significantly different from crizotinib (*p* > 0.05). Positive values in parentheses indicate a longer distance compared to crizotinib, while negative values indicate a shorter distance. A dash (-) indicates no interaction data available for the specific drug and residue pair.

**Table 4:** Average interaction distances of lead compounds with key residues of VEGFR-2 protein. This table presents the average interaction distances (in Ångströms, Å) between lead drug compounds and critical amino acid residues at the functional site of the VEGFR-2 protein, as depicted in Figure 4. These values, derived from molecular docking simulations, provide insights into the binding strength and specificity of each drug candidate. Shorter distances generally indicate stronger and more stable interactions, highlighting the potential efficacy of the compounds in targeting VEGFR-2. These data support the structural and functional analysis of the identified inhibitors.

Lead Dugs	Cys919 (SG) Å	Cys1024(SG) Å	Lys868 (NZ) Å	Asp1028 (OD2) Å	Asp1046 (OD2) Å
Cabozantinib	18.76 ± 0.34	8.94 ± 0.18	11.32 ± 0.06	10.78 ± 0.14	6.81 ± 0.17
Telmisartan	13.35 ± 0.12 *** (−5.41)	7.22 ± 0.10 *** (−1.72)	9.64 ± 0.11 *** (−1.68)	-	3.78 ± 0.04 *** (−3.03)
Entacapone	18.50 ± 0.07 (−0.26) ^NS^	4.22 ± 0.08 *** (−4.72)	10.02 ± 0.10 *** (−1.30)	-	-
Eltrombopag	19.68 ± 0.137 *** (+0.92)	16.54 ± 0.24 *** (+7.60)	11.02 ± 0.16 *** (−0.30)	-	-
Triamterene	20.27 ± 0.09 *** (+1.51)	-	17.20 ± 0.06 *** (+5.88)	3.84 ± 0.07 *** (−6.94)	8.89 ± 0.06 *** (+2.08)
Crizotinib	20.59 ± 0.11 *** (+1.83)	14.39 ± 0.10 *** (+5.45)	12.14 ± 0.20 *** (+0.82)	4.59 ± 0.07 *** (−6.19)	8.47 ± 0.12 *** (+1.66)
Dihydroergotamine	13.99 ± 0.05 *** (−4.77)	-	18.17 ± 0.05 *** (+6.85)	10.66 ± 0.04 (−0.12)	-
Fludarabine Phosphate	-	-	11.81 ± 0.26 *** (+0.49)	-	8.15 ± 0.16 *** (+1.34)

Note: A dash (-) indicates no interaction data available for the specific drug and residue pair. Values in parentheses represent the mean difference compared to cabozantinib. *** indicates *p* < 0.0001 (highly significant difference from cabozantinib). ^NS^ (not significant) indicates not significantly different from cabozantinib (*p* > 0.05). Positive values in parentheses indicate a longer distance compared to cabozantinib, while negative values indicate a shorter distance. A dash (-) indicates no interaction data available for the specific drug and residue pair.

## Data Availability

The original contributions presented in this study are included in the article. Further inquiries can be directed to the corresponding author(s).
